# Searching for Natural Conductive Fibrous Structures via a Green Sustainable Approach Based on Jute Fibers and Silver Nanoparticles

**DOI:** 10.3390/polym10010063

**Published:** 2018-01-11

**Authors:** Diana P. Ferreira, Armando Ferreira, Raul Fangueiro

**Affiliations:** 1 Centre for Textile Science and Technology (2C2T), University of Minho, 4800 Guimarães, Portugal; rfangueiro@civil.uminho.pt; 2 Center of Physics, University of Minho, 4710-057 Braga, Portugal; armando.f@fisica.uminho.pt

**Keywords:** natural fibers, jute fibers, silver nanoparticles, UV photoreduction, PEG, electrical conductivity

## Abstract

This paper provides new insights regarding jute fibers functionalization with silver nanoparticles (Ag NPs) with improved conductivity values and highlights the sustainability of the processes involved. These NPs were applied onto jute fabrics by two different sustainable methods: ultraviolet (UV) photoreduction and by using polyethylene glycol (PEG) as a reducing agent and stabilizer. Field Emission Scanning Electron Microscopy (FESEM) images demonstrated that the Ag NPs were incorporated on the jute fibers surface by the two different approaches, with sizes ranging from 70 to 100 nm. Diffuse reflectance spectra revealed the plasmon absorption band, corresponding to the formation of metallic Ag NPs, in all samples under study. Attenuated Total Reflectance-Fourier Transform Infrared Spectroscopy (ATR-FTIR) was used to characterize the obtained samples, demonstrating NPs adsorption to the surface of the fibers. The resistivity value obtained by the two-point probe method of the jute fabric without functionalization is about 1.5 × 10^7^ Ω·m, whereas, after NPs functionalization, it decreased almost 15,000 times, reaching a value of 1.0 × 10^3^ Ω·m. Further research work is being undertaken for improving these values, however, 1000 Ω·m of resistivity (conductivity = 0.001 S/m) is already a very reasonable value when compared with those obtained with other developed systems based on natural fibers. In summary, this work shows that the use of very simple methodologies enabled the functionalization of jute fibers with reasonable values of conductivity. This achievement has a huge potential for use in smart textile composites.

## 1. Introduction

Nowadays, the search for textiles and fibrous structures with the capacity of conducting electricity is in full growth because of the wide variety of potential applications of these fibrous structures, resulting from their flexibility, lightweight, and capability of adapting to different shapes [1,2,3]. Conductive fibrous systems can transport electrical signals as well as electrical current in order to be used as sensors for monitoring vital biological signs [4]. Their sensing functionalities make them suitable for several other application areas, such as the military sector, sports, engineering, therapeutics, and the automotive or aerospace industries [5,6]. Nowadays, synthetic fibers are still the most used type of fibers in industry, however, natural fibers, because of their low cost, abundance, biocompatibility, and biodegradability are very promising materials for replacing the synthetic ones [7]. Hence, a wide variety of different natural fibers, including jute fibers, are being used as reinforcements of composites fabrication [8,9,10,11].

Jute is highly available as it is the second most produced fiber in the world after cotton, because it is cheap, biodegradable, environmental friendly, and has a low density and good specific mechanical properties. Additionally, it can be woven into different forms and shapes [12,13]. However, jute and natural fibers are, in general, electrical insulators or present very limited conductivity. Therefore, it is a huge challenge to use natural fibers for smart conductive fibrous systems. One of the strategies that can be used to decrease the resistivity of natural fibers is their functionalization with conductive metallic elements [14,15,16]. Because of their size, metal nanoparticles (NPs) exhibit a high surface area and excellent surface activity which make them promising materials with exclusive optical, electrical, and catalytic properties [17,18,19]. One of the most used metal NPs for improving the conductivity and mechanical properties of coatings and composites are silver nanoparticles (Ag NPs) [20,21,22].

These Ag NPs can be synthesized by several different methods, including chemical reduction and UV photoreduction. They can be synthesized in a suspension and later adsorbed onto the fibers, or they can be produced in situ using UV radiation. The chemical reduction of Ag NPs comprises the use of a silver precursor, usually silver nitrate (AgNO_3_), relatively high temperatures, a reducing agent (sodium citrate, sodium borohydride, hydrazine, or hydroxylamine hydrochloride [23]), and, in some cases, a stabilizing agent to prevent nanoparticles aggregation. However, the use of high temperatures and highly toxic reducing or stabilizing agents decreases the process sustainability.

In order to overcome this issue, UV photoreduction of silver nanoparticles directly onto the fibers is considered a more environmental friendly method [24,25]. Another successful and more eco-friendly method for Ag NPs synthesis is the one using polyethylene glycol (PEG) [26]. PEG is one of the most used polymers for preventing the aggregation of NPs by the direct attachment of PEG molecules onto the NPs surface (PEGylation); however, PEG can also be used as both a reducing and a stabilizing agent for the formation of Ag NPs [17,26,27].

According to the literature review performed, only two research works mention the functionalization of jute fibers with silver nanoparticles, one regarding the in situ synthesis using silver nitrate at 90 °C [28], and another describing the Ag NPs synthesis by microwave heating on 2,2,6,6-tetramethylpiperidine-1-oxyl (TEMPO)-oxidized jute fibers [29]. For this reason, it was decided to take advantage of the abundantly available jute fibers and increase the sustainability of the processes involved in jute functionalization with Ag NPs even more. At the same time, and most importantly, it was decided to develop a new fibrous structure with promising values of electrical resistivity.

Hence, two sustainable and facile methodologies were applied for jute functionalization. The first one was the in situ green synthesis of Ag NPs onto jute fibers by the UV photoreduction process [30] in the absence of reducing reagents and only using an aqueous solution of a precursor (AgNO_3_) at room temperature. The second one was the rapid synthesis of the Ag NPs using PEG as the reducing agent without heating, followed by the impregnation of the jute with the synthesized NPs suspension. Also, instead of using hazardous fiber surface treatments like, for example, an alkaline treatment, plasma was used as the jute fabric pretreatment. Corona treatment is very effective as a pretreatment for fibers surfaces. In addition to surface “cleaning”, the plasma treatment promotes surface activation and improves the adhesion properties and surface roughness, leading to surfaces that are easier to functionalize without changing the whole material’s mechanical properties [31,32].

In this work, several parameters were evaluated to analyze the influence of each one on the final resistivity values of each sample: concentration of the precursor solution, jute samples’ immersion time, irradiation time in the photoreduction process, and PEG molecular weight.

Field Emission Scanning Electron Microscopy (FESEM) was used to analyze the NPs incorporation in the fabric, and chemical microanalyses of the samples were performed with the Energy Dispersive Spectroscopy (EDS) technique. To confirm the formation of the silver nanoparticles with PEG, Scanning Transmission Electron Microscopy (STEM) was used, as well as Energy Dispersive X-ray (EDX) Analysis. The formation of the metallic Ag plasmon was evaluated by Ground State Diffuse Reflectance spectroscopy (GSDR) in all the fabric samples under study. To evaluate the presence of the surface plasmon band correspondent to the metallic Ag NPs, in solution, UV–Visible spectroscopy was used. Fourier transformed infrared spectroscopy (FTIR) was utilized to characterize the obtained samples and evaluate jute functionalization with Ag NPs. The two-point probe method was applied for the evaluation of jute fabric electrical resistivity with and without NPs functionalization.

## 2. Materials and Methods

### 2.1. Materials

The jute fabric used in this work was supplied by RCS (Braga, Portugal) with an areal mass of 430 g/m^2^. Silver nitrate and Poly(ethyleneglycol) *M*r ~200, ~4000 and 16,000–24,000 were purchased from Sigma Aldrich (Algés, Portugal). The sample irradiation was performed with a UV blacklight lamp 15 W from HQ Power^TM^.

### 2.2. Sample Preparation

#### 2.2.1. Pretreatment of Jute Fabric with Plasma

The jute fabric was treated with corona plasma before nanoparticles functionalization. The discharge power utilized was 1 kW with a speed of 4 m/min. After 12 passages, each side with a dosage of 6 kW·min·m^−2^, the contact angle of the jute samples was measured.

#### 2.2.2. In Situ Synthesis of Silver Nanoparticles onto Jute

Samples of plasma-treated jute fabric (5 cm × 5 cm, average weight ~1.425 g) were immersed in 50 mL of AgNO_3_ aqueous solutions (0.01, 0.05, 0.1, and 0.2 M) for 2 h (optimized time), while ultrasound stirring each 20 min. After this, the excess of solution was discarded and the samples were placed under UV light for several irradiation times on each side (10 cm distance). After irradiation, the samples were washed with distilled water to remove any residue and dried at room temperature.

#### 2.2.3. Synthesis of Ag-PEG Nanoparticles and Deposition onto Jute

PEG aqueous solutions with different average molecular weights were used, i.e., 200, 4000, and 24,000, for the formation of silver nanoparticle colloids. PEG was used as a nontoxic reducing agent of AgNO_3_ precursor and at the same time acted as a dispersant and stabilizer. Several concentrations of AgNO_3_ onto PEG200/4000 and 24,000 aqueous solutions were made (0.01, 0.05, 0.1, and 0.2 M) and kept under stirring for 20–30 min at room temperature. When the solutions became yellow, the reduction of silver ions to silver metal (Ag^0^) was achieved, therefore the solutions were poured onto the jute fabric (5 cm × 5 cm, average weight ~1.425 g) and kept overnight. After this, the jute samples with the silver nanoparticles were centrifuged for removing the excess of the solution, washed with distilled water, and dried at room temperature or at 150 °C for 2 h.

### 2.3. UV–Visible Absorption Spectra

UV–Visible absorption spectra of the nanoparticles solutions were acquired using a Unicam double beam scanning UV–Visible spectrophotometer (ThermoFisher, Waltham, MA, USA) at room temperature in the spectral range from 190 to 1100 nm. Several aliquots were collected from the test samples and diluted to prevent deviation from Beer’s Law linearity.

### 2.4. Ground State Diffuse Reflectance and CIELAB Color Coordinates

The reflectance, *R*, from each sample was obtained with a Spectra Flash SF600 PLUS spectrophotometer supplied by Datacolor (Lucerne, Switzerland) in the spectral range from 360 to 700 nm. The remission function of the jute fabric functionalized with silver nanoparticles, *F*(*R*), was calculated using the Kubelka-Munk equation for optically thick samples: *F*(*R*) = (1 − *R*)^2^/(2*R*) = *K*/*S*, where *K* is the absorption coefficient and *S* the dispersion coefficient. Each sample was measured three times in different sites of the sample to ensure a relative homogeneity. CIELAB color coordinates were calculated with this Datacolor spectrophotometer using the difference Cielab coordinates D65/10 software (Lucerne, Switzerland) taking the untreated jute fabric as standard. The ΔL* shows the difference in lightness of the fabric; Δa* indicates if the sample is redder (if the values are positive) or greener (if negative); Δb* indicates the yellower color if positive and bluer if negative. Finally, the overall difference between the standard and the evaluated samples can be attributed to the ΔE* values calculated by the following equation: ΔE = [(ΔL*)^2^ + (Δa*)^2^ + (Δb*)^2^]^0.5^ [33].

### 2.5. Attenuated Total Reflectance-Fourier Transform Infrared Spectroscopy (ATR-FTIR)

ATR-FTIR spectroscopy studies were performed with an IRAffinity-1S, SHIMADZU equipment (Kyoto, Japan). Each spectrum was acquired in transmittance mode on a diamond ATR crystal cell by accumulation of 45 scans with a resolution of 8 cm^−1^ from 4000 to 380 cm^−1^. The samples were analyzed in different sites to ensure homogeneity.

### 2.6. Field Emission Scanning Electron Microscopy (FESEM) and Energy Dispersive X-ray (EDX)

Morphological analysis of the jute with nanoparticles was realized in an Ultra-high-resolution Field Emission Scanning Electron Microscopy (FESEM), NOVA 200 Nano SEM, FEI Company (Hillsboro, OR, USA). Before the analysis, the samples were covered with a very thin film (20 nm) of Au-Pd (80–20 weight %), using a high-resolution sputter coater, 208 HR Cressington Company (Watford, UK), coupled to a MTM-20 Cressington High Resolution Thickness Controller.

Secondary electron images, i.e., topographic images, were performed at an acceleration voltage of 10 kV. Atomic contrast images were realized with a Backscattering Electron Detector (BSED) (Hillsboro, OR, USA) at an acceleration voltage of 15 kV.

Chemical microanalyses of the jute samples were performed with the Energy Dispersive Spectroscopy (EDS) technique (Hillsboro, OR, USA), using an EDAX Si (Li) detector at an acceleration voltage of 15 kV.

The solution of PEG containing silver nanoparticles was analyzed in transmission electron mode, the samples were installed in Cu-C grids by immersion in the solution. After this, the samples were analyzed with an acceleration voltage of 15 kV, using a scanning transmission electron detector (STEM) (Hillsboro, OR, USA). A Zeiss Ultra 55 FESEM (Hillsboro, OR, USA) was used to observe the morphology of the samples using an acceleration voltage of 3 kV. Energy dispersive X-ray (EDX) measurements were performed between 0 and 10 kV.

### 2.7. Resistivity Measurements Using the Two-Point Probe Method

The electrical resistivity of the samples was measured by the two-wire method, where the voltage was applied, and the current measured by a Keithley 487 picometer/voltage source (Tektronix, OR, USA). All measurements were performed in direct current (DC) mode, at room temperature. The measurements were carried out in air using custom-made copper tips. The procedure was carried out under a cover, to achieve a dark environment. Humidity and cleanness were considered as constant. The attachment of the contacts was checked prior to every run (I/V correlation close to 1) to ensure that an ohmic contact was obtained. The electrical resistivity ρ (Ω·m) was calculated by:

(1) $$\rho =R\frac{A}{L}$$

where *R* is the electrical resistance, *A* is the area of the electrode (5 mm × 1 mm), and *L* is the distance between the electrodes (3 mm). The electrical conductivity is given by the inverse of the electrical resistivity 1/ρ.

## 3. Results and Discussion

### 3.1. Jute Fabric Pretreatment

It is well known that plasma treatment is a widely used and very successful technique in the surface treatment of natural fibers. Several works describe the effect of plasma on jute fibers: the morphology and hydrophilicity character of the surface changes, the fact that the surface becomes rougher, thereby increasing the compatibility with polymeric materials, and the increase in wickability [34,35,36]. At the same time, the corona process can induce the oxidation of the material’s surface by the oxidants produced in corona discharge (ozone, atomic oxygen, and oxygen free radicals). These oxidants, combined with the free radicals of the material surface, can create oxidizing groups, for example, carboxyl groups. The introduction of these polar groups on the material’s surface could increase the surface energy, improving the wettability and adhesion properties of the materials [37]. The presence of these carboxyl groups can also be very important for nanoparticles formation: some works describe the need to use oxidizing agents like TEMPO (2,2,6,6-tetramethylpiperidine-1-oxyl radical) in order to facilitate the reduction of the silver nanoparticles [29,38]. Furthermore, the process can also be used for cleaning the surface of the material, for example, to remove waxes [39], leaving the cellulosic groups more available for nanoparticles nucleation.

Taking all of this into consideration, it was decided to use the corona treatment instead of any hazardous chemical treatment to facilitate surface functionalization with Ag NPs. Raw jute fibers are mainly composed of cellulose (61–71%), followed by hemicellulose (13–20%), lignin (12–13%), and waxes (0.5%) [40], and are hydrophilic in nature. However, because of the presence of some waxes, fat, and pectin in the external layer of the fibers used in this work, the measured contact angle was about 152.5°. The application of the corona pretreatment drastically decreased the jute fabric’s contact angle from ~152.5° to approximately 0°, turning the surface hydrophilic and allowing and facilitating the reaction in aqueous medium. Furthermore, and as referred before in the introduction, the mechanical properties of the material were not affected by the plasma treatment. For example, the value of the tensile strength obtained for the untreated jute was 1323 N and for the plasma-treated jute was 1332 N.

### 3.2. In Situ Jute Functionalization by the UV Photoreduction Process and Resistivity Values

**Figure 1:**
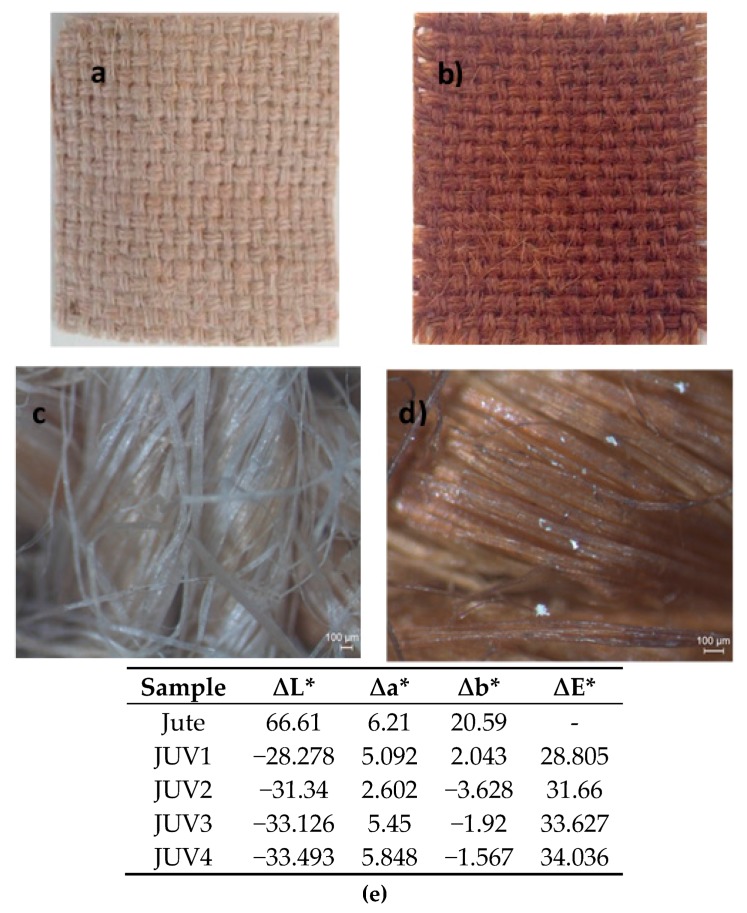
(**a**,**c**) Image of the jute fabric without functionalization; (**b**,**d**) jute fabric functionalized with silver nanoparticles. (**e**) CIElab color coordinates values.

The in situ green synthesis of Ag NPs on jute fibers by the UV photoreduction process was performed using only the AgNO_3_ aqueous solution at room temperature. In this method, the glucose monomers from cellulose are responsible for the reduction of the Ag^+^ ions. Concretely, UV radiation will break the oxygen bridges between the glucose monomers in the cellulose structure of the jute allowing the formation of aldehyde groups that could reduce the Ag^+^ ions. In this way, the use of hazardous chemical reducing agents and high temperatures for silver nanoparticles reduction was avoided. The color change, confirmed by the CIElab coordinates, from pure jute’s colour (beige) to brown, as can be seen in Figure 1, indicates the nanoparticles reduction onto the jute fabric, confirmed in the next section with the samples characterization by GSDR, FESEM, EDS, and FTIR. The images obtained with a stereo microscope at a higher magnification allowed us to see the differences between the jute fabric (c) and the jute-Ag NPs, characterized by a darker color and exhibiting some bright areas, characteristic of Ag nanoclusters. The CIElab color coordinates exhibited in Figure 1 were obtained for the samples described in Table 1, using the jute fabric as standard and evaluating the CIElab difference coordinates between the jute and the jute with Ag NPs (samples JUV1 to JUV4). The ∆L* values decreased from the jute to the samples with Ag NPs, indicating that the samples became darker. At the same time, the ∆E* values increased, corroborating an overall color difference between the untreated jute and the treated jute samples (JUV1 to JUV4). In general, the samples became redder and bluer accordingly with the Δa* and Δb* parameters.

For this UV method, several samples were prepared varying the AgNO_3_ solution concentration, the jute immersion time in this solution, and the UV irradiation time, as Table 1 shows.

**Figure 2:**
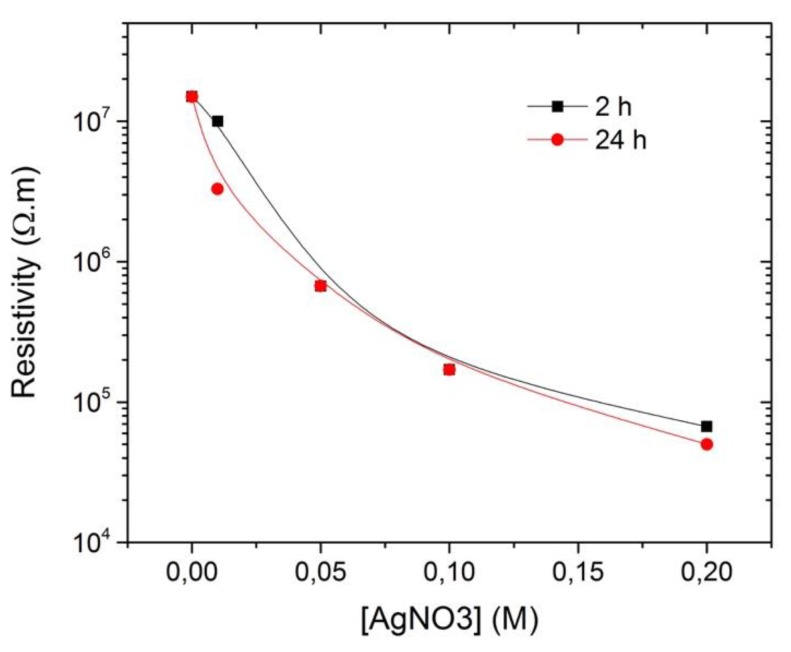
Graphic demonstrating the resistivity values dependence on the precursor concentration.

As referred before, the main goal of this work was to decrease the resistivity values of the jute fabric with the functionalization of Ag NPs. Hence, all the modifications in the synthesis protocols were made accordingly with the values obtained for resistivity. As it can be observed in Table 1, using the UV method, several samples were obtained utilizing four different concentrations, from 0.01 M to 0.2 M; the jute immersion time in the precursor aqueous solution varied from 2 to 24 h, and the jute samples were irradiated on each side from 5 to 10 h. From the Jute sample to the JUV4 (0.2 M), the resistivity values decreased with the concentration of the precursor. The jute fabric has a very high value of resistivity (1.5 × 10^7^ Ω·m) which decreased drastically to 6.67 × 10^4^ Ω·m when it was functionalized with silver nanoparticles using a precursor concentration of 0.2 M, as can be clearly observed in Figure 2. Also, when comparing JUV4 with JUV8, obtained using the same precursor concentration but varying the irradiation (5 to 10 h) and immersion times (2 to 24 h), no significant changes were observed in the resistivity values, as presented in Figure 2, leading to the conclusion that these parameters have little influence on the final resistivity values.

### 3.3. Jute with Ag-PEG NPs and Resistivity Values

The second method used for Ag NPs synthesis was the chemical reduction using PEG as a reducing agent and stabilizer. PEG is a nontoxic, biodegradable polymer [41] widely used in the synthesis of silver nanoparticles colloids [42,43,44]. Because of the high binding affinity between the PEG hydroxyl groups and Ag ions, the Ag^+^ ions can form complexes with the PEG molecules through a direct redox reaction between PEG and AgNO_3_. The Ag^+^ ions can be progressively reduced to Ag^0^ through the oxidation of the hydroxyl groups in PEG: CH_2_CH_2_OH + Ag ^+^ → CH_2_CHO + Ag^0^ [23,45].

**Figure 3:**
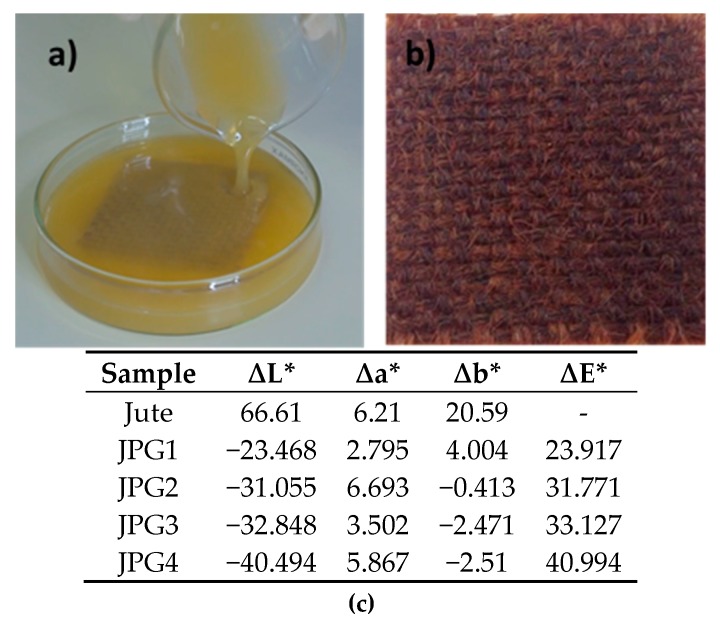
(**a**) Jute fabric impregnated with Ag^0^-PEG suspensions; (**b**) jute fabric with Ag^0^-PEG NPs; (**c**) CIElab color coordinates.

For this purpose, several suspensions of Ag NPs with different concentrations of the precursor were developed, in which the jute was subsequently immersed for 24 h. Surprisingly, and contrary to the already published papers, there was no need to heat the solutions in order to obtain Ag NPs (Figure 3a). With PEG200, it was possible to obtain Ag NPs colloids by using AgNO_3_ in the PEG solution at room temperature and with sunlight. The obtained yellow color suspension (indicating silver reduction, presented in Figure 3a) was used for jute impregnation, resulting in dried jute fabric functionalized with silver nanoparticles (brown sample, Figure 3b), as will be demonstrated in the following sections with the FESEM, EDS, GSDR, and FTIR analyses. Like the previous samples obtained with the UV method, the jute fabric became darker following the impregnation with Ag NPs, as indicated by the CIElab parameters presented in Figure 3.

PEG with different molecular weights was used (200, 4000, and 24,000), and a sintering process was included at 150 °C for 2 h. In the study performed by Kardarian et al., it was shown that heating samples of cotton coated with silver nanoparticles at 200 °C for 2 h increased their electrical conductivity [46], therefore it was decided to test this phenomenon in the samples developed in this work composed by jute-Ag NPs at 150 °C. In fact, by observing Table 2 and comparing the sample JPG5 with JPG1, obtained by using the same AgNO_3_ concentration (0.01 M), it is evident that the use of 150 °C decreased the resistivity value from 3000 to 1670 Ω·m, and consequently increased the conductivity.

**Figure 4:**
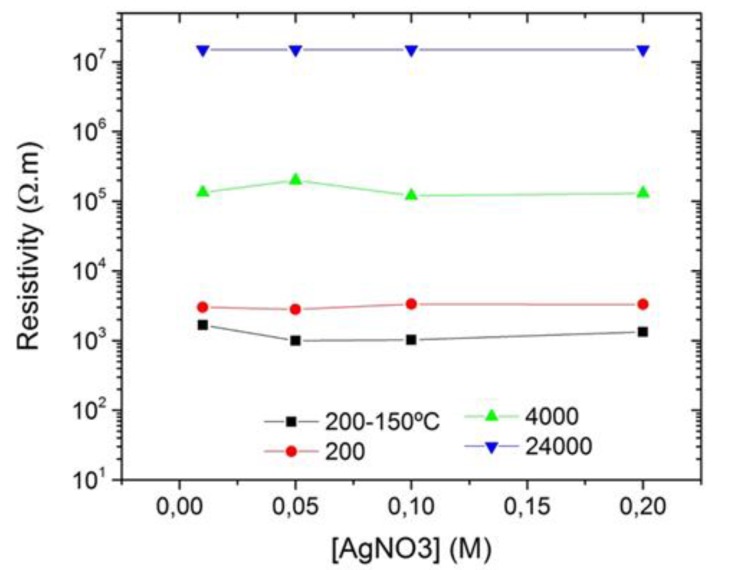
Dependence of the resistivity values on AgNO_3_ concentration.

Besides the temperature, it was expected that the precursor concentration had a pronounced effect on the resistivity values, therefore AgNO_3_ concentration was increased from 0.01 to 0.2 M. When comparing all the samples obtained with different AgNO_3_ concentrations, no changes were detected in the resistivity values by using an AgNO_3_ initial concentration of either 0.01 or 0.2 M, as can be seen in Figure 4. The PEG hydroxyl groups are responsible for silver reduction, as it was already described, and, at the same time, PEG acts as a dispersant, and protects the formed Ag NPs from aggregation by forming a coating around them, as observed in the STEM images presented in the following section. In this case, a suspension composed of Ag NPs coated and protected with PEG groups will be obtained. Because of the presence of the hydroxyl groups of cellulose (from the jute), the expected trend will be the formation of hydrogen bonds between cellulose and the PEG groups, which will ultimately improve the incorporation of the Ag-PEG NPs on the jute fabric. These hydrogen bonds can form as long as there are hydroxyl groups available in the jute fabric. The attachment of Ag NPs will be hindered as soon as the cellulosic groups are completely occupied. Jute fibers will only be functionalized with Ag NPs accordingly with the minimum required amount of AgNO_3_ to form hydrogen bonds with cellulose. Therefore, the final values of jute resistivity do not change with increasing AgNO_3_ concentrations, as observed in Table 2.

Regarding the use of PEG 4000, no significant changes were detected, and the best conductivity results were obtained with PEG 200. PEG 24,000 was also used for silver reduction, but there was no nanoparticles formation after 1 h of reaction, possibly indicating the need of using a heating process at a higher temperature, as some works describe [44,47].

In addition to the obtained resistivity values, the characterization of the jute fabric, together with the synthesized nanoparticles, becomes essential. It should be shown that Ag NPs formed and that the jute fabric was functionalized with them. Thus, several characterization techniques were utilized, including UV–Vis spectroscopy, GSDR, FESEM, STEM, EDS, EDX, and ATR-FTIR.

### 3.4. Characterization of Silver Nanoparticles Produced with PEG

#### 3.4.1. UV–Visible Spectroscopy

**Figure 5:**
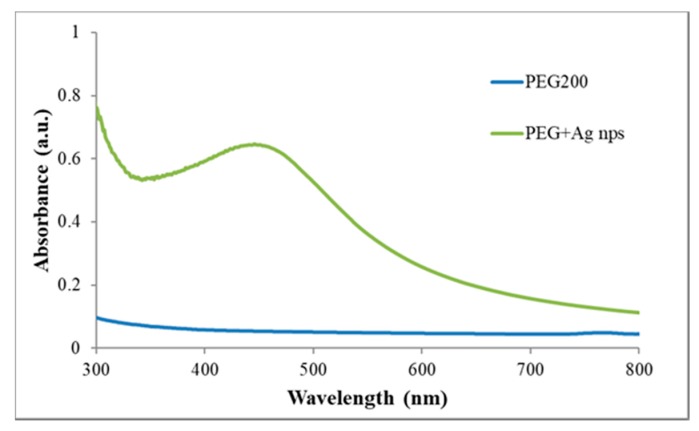
Absorption spectra of a PEG solution and of a solution with PEG-Ag NPs obtained using 0.1 M AgNO_3_.

By using UV–Visible spectroscopy, it is possible to obtain information about Ag NPs formation with PEG solutions and their average particle size. Figure 5 shows the absorption spectrum of a PEG aqueous solution without NPs compared with the spectrum of the colloid composed of Ag-PEG NPs, obtained using 0.01 M AgNO_3_ stirred with PEG200 at room temperature for 20 min (impregnation solution of the JPG3 sample).

In the spectrum of the Ag-PEG NPs solution, it is visible the absorption band characteristic of Ag NPs formation, resulting from the surface plasmon resonance (SPR) of Ag NPs peaking at approximately 456 nm, with a full width at half maximum (FWHM) of ~125 nm. The maximum absorption value of these synthesized nanoparticles indicates the existence of particles with sizes ranging from 70 to 100 nm [48]. Whereas the FWHM gives us information about the dispersion of the NPs in the solution, values extended over 100 nm indicate NPs polydispersion in the solution. This polydispersion is also visible from the broadening of the plasmon band, indicating the possible formation of Ag NPs agglomerates or nanoclusters, as it was observed in the STEM analysis.

#### 3.4.2. Scanning Transmission Electron Microscopy (STEM)

**Figure 6:**
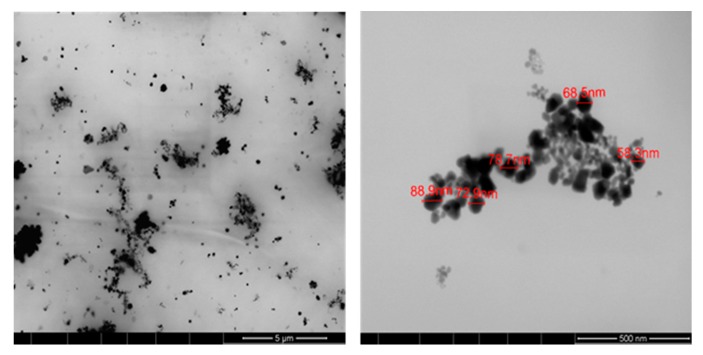
STEM micrographs of the Ag NPs-PEG solution with different magnifications: 5 µm and 500 nm.

The size and morphology of AgNPs synthesized with PEG200 was evaluated using STEM, as shown in Figure 6. As can be seen in the micrographs obtained, Ag NPs were successfully synthesized using PEG200, exhibiting polydispersion in the PEG medium and different sizes varying from 60 to 90 nm, in accordance with the results obtained by UV–Vis spectroscopy. As mentioned before in Section 3.3, in the selected images, the PEG coating can be seen around the Ag NPs, characterized by the connecting polymeric “wires” around and between nanoparticles.

**Figure 7:**
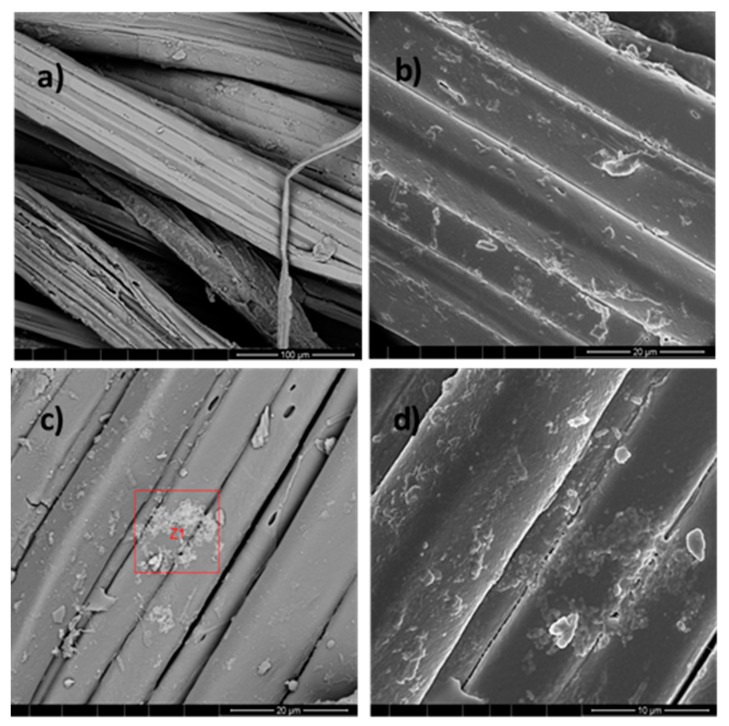
Images of jute fabric impregnated with Ag NPs (sample JPG3) synthesized with PEG, with different magnifications: (**a**) 100 µm, (**b**,**c**) 20 µm, and (**d**) 10 µm. The (**b**,**c**) images are in topographic mode, and the Z1 area was used for the EDS analysis.

After demonstrating Ag NPs formation, these NPs were used for jute fabric impregnation, and the final samples, composed of jute fabric with Ag NPs, were analyzed by FESEM. Figure 7 shows, as an example, the images obtained for the sample obtained with 0.1 M AgNO_3_ (JPG3). The images, obtained by using 10 and 20 µm of resolution in the topographic mode (Figure 7b,d), clearly show the NPs distribution all over the jute fibers.

**Figure 8:**
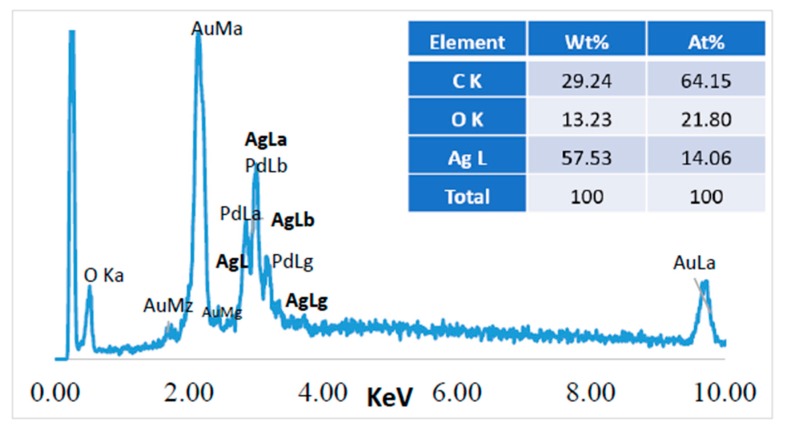
Chemical microanalysis of the jute fabric surface with Ag NPs (sample JPG3), EDS spectrum, and elemental composition.

Energy Dispersive Spectroscopy (EDS) was performed in the Z1 area (20 µm^2^) indicated in Figure 7c, and the results obtained for the jute surface characterization are presented in Figure 8.

Before performing a chemical microanalysis, the samples were covered with a very thin film of Au-Pd, which originated the high intensity peaks from Au and Pd observed in Figure 8. Carbon and oxygen are constituent elements of the cellulosic fibers structure, therefore they are also abundantly present in the jute surface. The presence of silver nanoparticles on the surface of jute was confirmed by the additional strong peak at ~2.98 keV, corresponding to the AgLa family of spectral lines. The amount of this element was obtained by EDS, indicating 57% of silver on the jute fabric’s surface. All the FESEM images together with the EDS analysis could confirm the incorporation of the Ag NPs onto the jute fabric surface, however, GSDR could also give us additional information about the presence of the NPs onto the fabric, as the next section will show.

#### 3.4.3. Ground State Diffuse Reflectance

**Figure 9:**
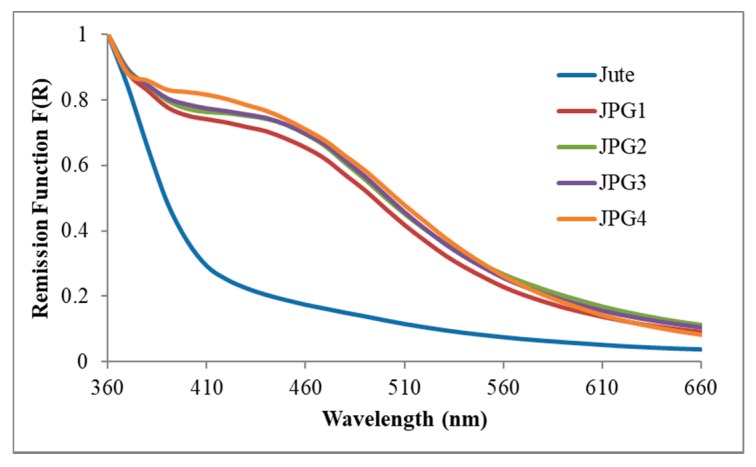
Normalized GSDR spectra of the jute fabric with Ag NPs-PEG obtained using different AgNO_3_ concentrations.

Figure 9 presents the Kubelka-Munk remission function for the jute fabric and the different samples of jute fabric with silver nanoparticles.

In the presented wavelength range, all the samples showed a band peaking at ∼460 nm, resembling the absorption obtained in solution for the silver surface plasmon (Figure 5). As the AgNO_3_ precursor concentration increased from 0.01 M (sample JPG1) to 0.2 M (JPG4), a slight increase in the plasmon band intensity was observed. Even so, the absorption bands of all samples were very similar in form and intensity, corroborating the hypothesis described earlier about the low influence of AgNO_3_ concentration in the final samples of jute with Ag NPs. Therefore, whatever the precursor concentration under use, we will have Ag NPs onto the jute surface until the extinction of possible anchoring sites.

### 3.5. Characterization of the Silver Nanoparticles Synthesized In Situ onto Jute Fabric

**Figure 10:**
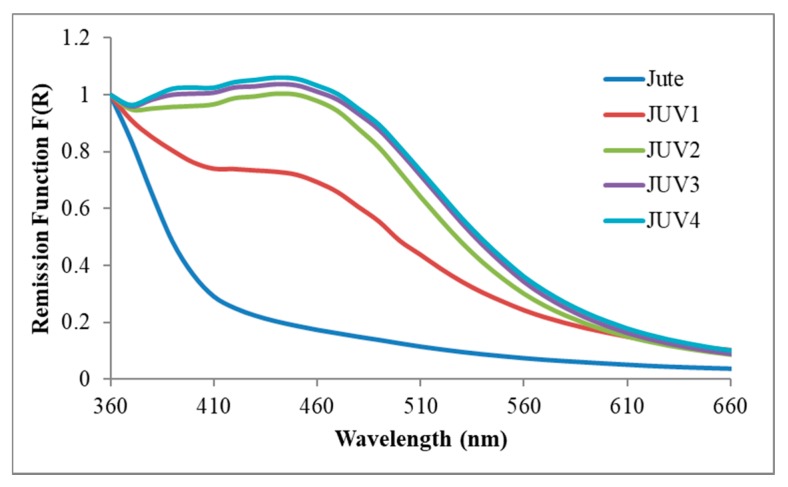
Normalized GSDR spectra of the jute fabric with Ag NPs using the UV photoreduction method and different AgNO_3_ concentrations.

The techniques used until this section were also applied for the characterization of the jute fabric with Ag NPs obtained by the UV photoreduction method. As already demonstrated for the PEG method, the GSDR spectra of Figure 10 also show the plasmon band which corresponds to the Ag NPs onto the jute surface. The plasmon band intensity increased with the increase of AgNO_3_ concentration until 0.2 M. This, together with the different resistivity values obtained with each concentration used, from 1.0 × 10^7^ to 6.67 × 10^4^ Ω·m, indicates that, contrary to the PEG method, in the photoreduction method the precursor concentration has some influence.

**Figure 11:**
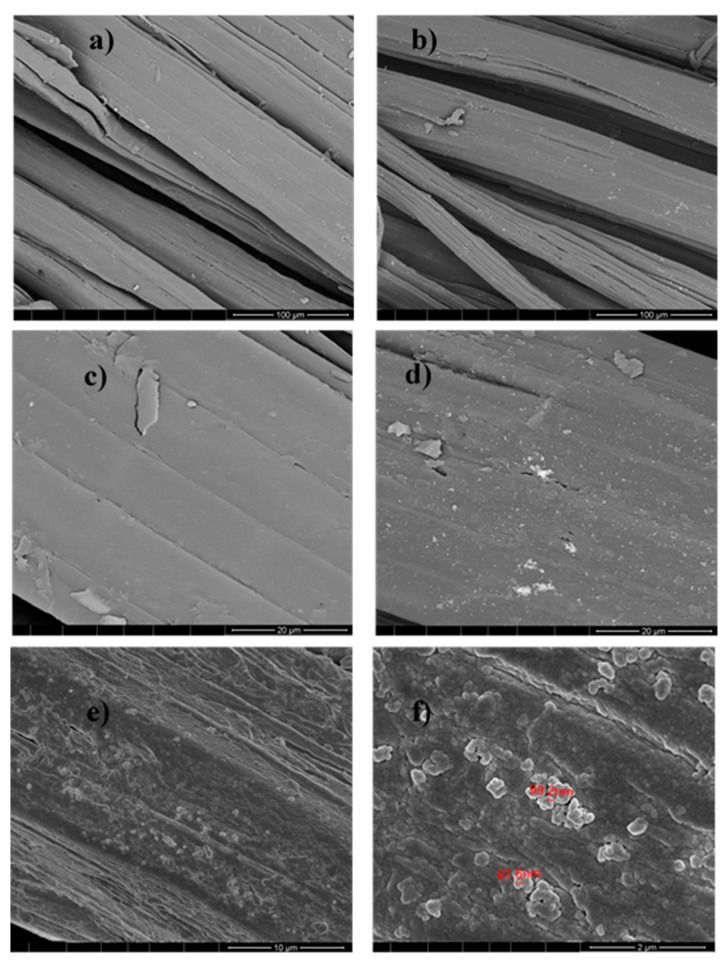
FESEM analysis of jute without NPs (**a**,**c**), and sample JUV2 (**b**,**d**,**e**,**f**) with different magnifications: (**b**) 100 µm, (**d**) 20 µm, (**e**) 10 µm, and (**f**) 2 µm. The (**e**,**f**) images are in topographic mode.

**Figure 12:**
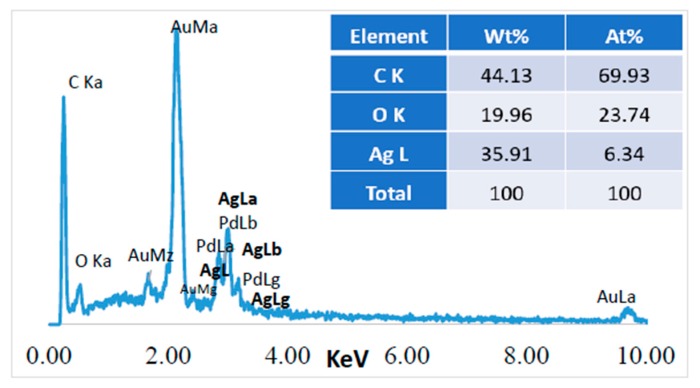
Chemical microanalysis of the jute fabric with Ag NPs obtained with the UV method, using JUV2 sample as example.

FESEM and EDS analyses were performed for all samples obtained by the UV photoreduction method, and the results from JUV2 sample are shown as an example in Figure 11 and Figure 12.

The morphological study from Figure 11 performed by FESEM gave information about the jute fibers’ surface with and without Ag NPs. Comparing (a) with (b), and (c) with (d) using 100 and 20 µm respectively, it is visible the appearance of the silver nanoparticles clusters onto the jute surface, characterized by the “bright” areas where the EDS analysis was achieved. Regarding Figure 11e,f showing topographic analyses, the Ag NPs appeared well distributed onto the jute surface, with sizes between 87 and 100 nm. Their presence was also proved by the elemental analysis of Figure 12, which indicated 36% of silver as an element in the sample under study.

### 3.6. ATR-FTIR Characterization

**Figure 13:**
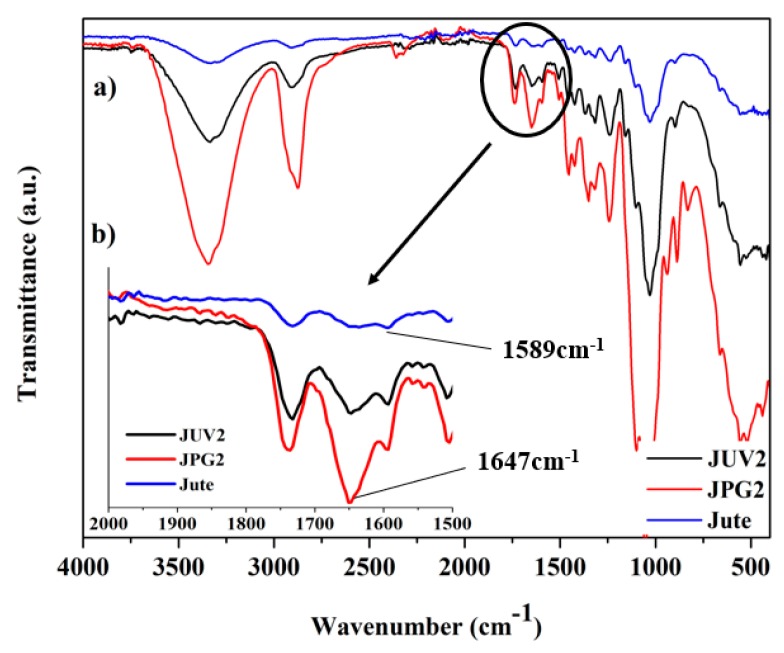
ATR-FTIR spectra of the jute fabric, sample JUV2, and JPG2.

ATR-FTIR spectroscopy was used to characterize the obtained samples, compare the different applied methods, and study the possible interactions between the materials. As examples, the FTIR spectra of the jute fabric, sample JUV2, and JPG2 are presented in Figure 13. In (a), it is presented the complete spectra ranging from 4000 to 400 cm^−1^, and in (b) the zoomed spectra from 2000 to 1500 cm^−1^ for the three different samples.

The ATR-FTIR spectrum of the jute fabric without any functionalization showed the typical assignments from the cellulosic-based fibers, namely, the bands from cellulose, hemicellulose, and lignin. Some of the most important bands were discriminated to compare the different samples under study, obtained using the two different methods. The band peaking around 3350 cm^−1^ arises from the O–H stretching vibration of cellulose and lignin, the band at 2900 cm^−1^ is the asymmetrical C–H stretching vibration of cellulose and hemicellulose, the band at approximately 1732 cm^−1^ is the C=O stretching vibration of the hemicellulose and lignin, while the aromatic C=C stretching from lignin is at ~1643 cm^−1^ [49,50,51]. All the described bands were present in both samples obtained by the two different methods, as can be seen in the general spectra.

Additionally, and considering the spectrum of jute with A-PEG NPs (JPG2), beyond the jute characteristic bands, three distinct fingerprint peaks of PEG could be observed: 834, 941, and 1246 cm^−1^. The absorption bands at 1342 and 2883 cm^−1^ were also from PEG, corresponding to the bending vibration and stretching vibration of the C–N bond [41]. When comparing the jute spectrum with the jute Ag-PEG NPs, an increase of band intensity between 3200–3500 cm^−1^ was visible, corresponding to the stretching vibrations of O–H and N–H bonds. This increase could indicate intermolecular hydrogen bonding formed between the groups from jute and the hydroxyl groups from PEG, as it was expected from the results presented before.

When comparing the three samples, it is evident that the most pronounced effect was related with the zoomed area of Figure 13b. The band peaking at ~1643 cm^−1^ appeared in both samples functionalized with silver nanoparticles at 1647 cm^−1^ with increased intensity. Also, the 1589 cm^−1^ peak was more perceptible in the samples with Ag NPs at 1593 cm^−1^ (JUV2) and 1600 cm^−1^ (JPG2). Cao et al. described the presence of an absorption band peaking at 1594 cm^−1^, attributed to the carbonyl groups resulting from jute surface oxidation [29], and Fahmy et al. referred that also the band peaking at 1647 cm^−1^ [42] corresponded to the carbonyl groups formation. Therefore, we suggest that the intensity increase in this area of the spectra for the jute sample functionalized with Ag NPs was due to carbonyl group formation resulting from the oxidation of hydroxyl groups by the Ag^+^ ions, attesting the reduction of Ag^+^ to Ag^0^ metal on the jute surface.

## 4. Conclusions

Two different sustainable methods were successfully applied for jute surface functionalization with silver nanoparticles. The UV photoreduction method was used in an aqueous medium and in the absence of any hazardous chemicals or heating process. With this method, several samples of Ag NPs deposited onto jute fabric were developed by varying some reaction parameters; the FESEM analysis along with the EDS, GSDR, and ATR-FTIR analyses, confirmed the existence of Ag nanoparticles on the surface of jute fabric, with sizes ranging from 87 to 100 nm. Simultaneously, jute resistivity decreased from 1.5 × 10^7^ to 5.0 × 10^4^ Ω·m following NPs deposition, reaching 0.0001 S/m of conductivity.

With the second method, using PEG as a reducing agent and a stabilizer, the adsorption of Ag NPs onto the jute fabric was also successfully achieved, as all the characterization methods demonstrated. With this process, it was possible to obtain samples with 1000 Ω·m of resistivity (conductivity = 0.001 S/m), decreasing more than 15,000 times compared with jute’s initial resistivity values. We are still working on improving these values, however, a conductivity = 0.001 S/m is already a very reasonable value when compared with the conductivity values obtained with other developed systems based on natural fibers, like the Ag NPs-polypyrrole–cotton fabric with σ = 0.48 S/m [52], or the Ag NPs-cotton with σ = 0.002 S/m [53]. The comparison is made with cotton because there is no work in the literature mentioning conductivity increases of the jute fabric with silver NPs. Therefore, for the first time, the jute-Ag NPs fabric was characterized considering its conductivity.

Overall, this work demonstrates two simple, economical, and functional processes to obtain conductive natural fibers to be used in a variety of monitoring and sensing applications. Because of the well-known antimicrobial and antibacterial properties of Ag NPs, we are also sure that these materials could be used with that purpose.

Currently, we are already working on the functionalization of other natural fibers with Ag NPs with higher conductivity values, for the development of new materials composed of these fibers and of biopolymers, and we believe that we will obtain very promising results in the very near future.

## Figures and Tables

**Table 1:** Different samples prepared by the UV photoreduction process with variations in the concentration of the precursor, immersion time, and UV irradiation time.

Sample	[AgNO_3_] M	Immersion time	Irradiation time (each side)	Resistivity Ω·m
Jute	0	0	0	1.5 × 10^7^
JUV1	0.01	2 h	5 h	1.0 × 10^7^
JUV2	0.05	2 h	5 h	6.7 × 10^5^
JUV3	0.1	2 h	5 h	1.7 × 10^5^
JUV4	0.2	2 h	5 h	6.7 × 10^4^
JUV5	0.01	24 h	10 h	3.3 × 10^6^
JUV6	0.05	24 h	10 h	6.7 × 10^5^
JUV7	0.1	24 h	10 h	1.7 × 10^5^
JUV8	0.2	24 h	10 h	5.0 × 10^4^

**Table 2:** Samples of jute with Ag NPs synthesized with PEG and the respective resistivity and conductivity values.

Sample	[AgNO_3_] M	PEG molecular weight	Impregnation time	Sintering process	Resistivity Ω·m
Jute	0	0	0	-	1.50 × 10^7^
JPG1	0.01	200	24 h	150 °C/2 h	1.67 × 10^3^
JPG2	0.05	200	24 h	150 °C/2 h	1.00 × 10^3^
JPG3	0.1	200	24 h	150 °C/2 h	1.02 × 10^3^
JPG4	0.2	200	24 h	150 °C/2 h	1.33 × 10^3^
JPG5	0.01	200	24 h	-	3.00 × 10^3^
JPG6	0.05	200	24 h	-	2.80 × 10^3^
JPG7	0.1	200	24 h	-	3.33 × 10^3^
JPG8	0.2	200	24 h	-	3.30 × 10^3^
JPG9	0.01	4000	24 h	-	1.33 × 10^5^
JPG10	0.05	4000	24 h	-	2.00 × 10^5^
JPG11	0.1	4000	24 h	-	1.20 × 10^5^
JPG12	0.2	4000	24 h	-	1.30 × 10^5^
JPG13	0.01	24,000	24 h	-	1.50 × 10^7^
JPG14	0.05	24,000	24 h	-	1.50 × 10^7^
JPG15	0.1	24,000	24 h	-	1.50 × 10^7^
JPG16	0.2	24,000	24 h	-	1.50 × 10^7^
